# Lupus nephritis: The regulatory interplay between epigenetic and MicroRNAs

**DOI:** 10.3389/fphys.2022.925416

**Published:** 2022-09-16

**Authors:** Ning Xu, Jie Liu, Xiangling Li

**Affiliations:** ^1^ School of Clinical Medicine, Affiliated Hospital of Weifang Medical University, Weifang, China; ^2^ Department of Nephrology, Affiliated Hospital of Weifang Medical University, Weifang, China

**Keywords:** systemic lupus erythematosus, lupus nephritis, histone modifications, noncoding RNAs, epigenetic

## Abstract

MicroRNAs (miRNAs) are endogenous, small, non-coding RNA molecules that act as epigenetic modifiers to regulate the protein levels of target messenger RNAs without altering their genetic sequences. The highly complex role of miRNAs in the epigenetics of lupus nephritis (LN) is increasingly being recognized. DNA methylation and histone modifications are focal points of epigenetic research. miRNAs play a critical role in renal development and physiology, and dysregulation may result in abnormal renal cell proliferation, inflammation, and fibrosis of the kidneys in LN. However, epigenetic and miRNA-mediated regulation are not mutually exclusive. Further research has established a link between miRNA expression and epigenetic regulation in various disorders, including LN. This review summarizes the most recent evidence regarding the interaction between miRNAs and epigenetics in LN and highlights potential therapeutic and diagnostic targets.

## Introduction

Systemic lupus erythematosus (SLE) is a severe and recurrently progressive autoimmune disease that manifests clinically with various symptoms, including fatigue, rash, joint pain, and kidney damage (Olson et al., 2022). Lupus nephritis (LN) is a common complication of SLE in many individuals, and the treatment of advanced stages of LN is still unclear.

Recently, LN treatment has primarily been determined by the histological class of the disease. Classes I and II usually do not require treatment, unlike Classes III and IV. The treatment of Classes V and VI remains contentious (Aziz and Chaudhary, 2018). Non-immunosuppressive therapies such as renin-angiotensin-aldosterone system (RAAS) blockade with angiotensin-converting enzyme inhibitors, angiotensin receptor blockers (ARBs), anticoagulation therapy, and lipid-lowering therapy have been used to treat LN. However, Immunosuppressive therapy, such as the administration of cyclophosphamide, calcineurin inhibitors (CNI), tacrolimus, and cyclosporine A (CSA), does not produce the desired results (Hahn et al., 2012).

Clinical trials to target pathways other than classical pathways of complement systems, including the lectin pathway and alternative pathway, have also been conducted (Anders et al., 2020; Villanueva et al., 2022). The anti-inflammatory and immunomodulatory properties of stem cells in LN are currently being investigated in clinical trials. Villanueva et al. (2022) demonstrated that CD11b agonists offer a novel approach to treating LN. Similarly, Olson et al. (2022) found that DAMPs with nucleic acid scavengers are promising for treating autoimmune disorders. Apart from medication changes, lifestyle changes can be made to avoid triggers, reduce damage and inflammation, and alleviate symptoms (Fava and Petri, 2019; Seet et al., 2021). While all these therapies have shown some promise in delaying disease progression or onset, their application remains in the early stages of development. Many factors, such as metabolic disorders, inflammation, immunoregulation, genetic disposition, and the environment, all play a role in the development of LN (Fava and Petri, 2019; Seet et al., 2021; Villanueva et al., 2022); however, the exact mechanism and safety of effective treatments must be explored *via* additional high-quality studies.

Recent advances in epigenetics have advocated its crucial role in regulating the physiological and pathological processes associated with LN development and progression (Zheng et al., 2021). Epigenetics is the study of gene expression and function and the generation of heritable phenotypes without modifying DNA sequences (Zheng et al., 2021; Zhang et al., 2022). Recent evidence has established that microRNA (miRNA), as an epigenetic factor, plays a critical role in the onset and development of various diseases, including cancer, cardiometabolic illnesses, inflammatory disorders, renal disorders, and hepatitis C, and several clinical trials are currently underway to develop therapeutic miRNAs as the next line of medical treatment (Zhang et al., 2022).

miRNAs function by silencing specific messenger RNAs, thereby regulating gene expression at the post-transcriptional level. Most miRNAs are transcribed from specific DNA sequences as primary (pri)-miRNAs and then processed to form pre-miRNAs and finally, mature miRNAs (Kaur et al., 2022). Although the potential mechanism remains unknown, some studies have demonstrated the critical role of epigenetic modulation in the regulatory circuit between epigenetic modulation and miRNAs, as miRNA genes can be epigenetically regulated by DNA methylation or histone modification. As a result, a subclass of miRNAs dubbed “epi-miRNAs” was identified that directly targets epigenetic regulators, such as DNA methyltransferases (DNMTs) (Turchinovich et al., 2013; Ichii and Horino, 2018). Few studies have been published on epigenetic-based drugs for clinical treatment. Hence, this review examines and describes the complex regulatory relationship between miRNAs and epigenetics, as well as the role of miRNAs in the development and progression of LN.

## Biogenesis and characteristics of microRNAs

**FIGURE 1:**
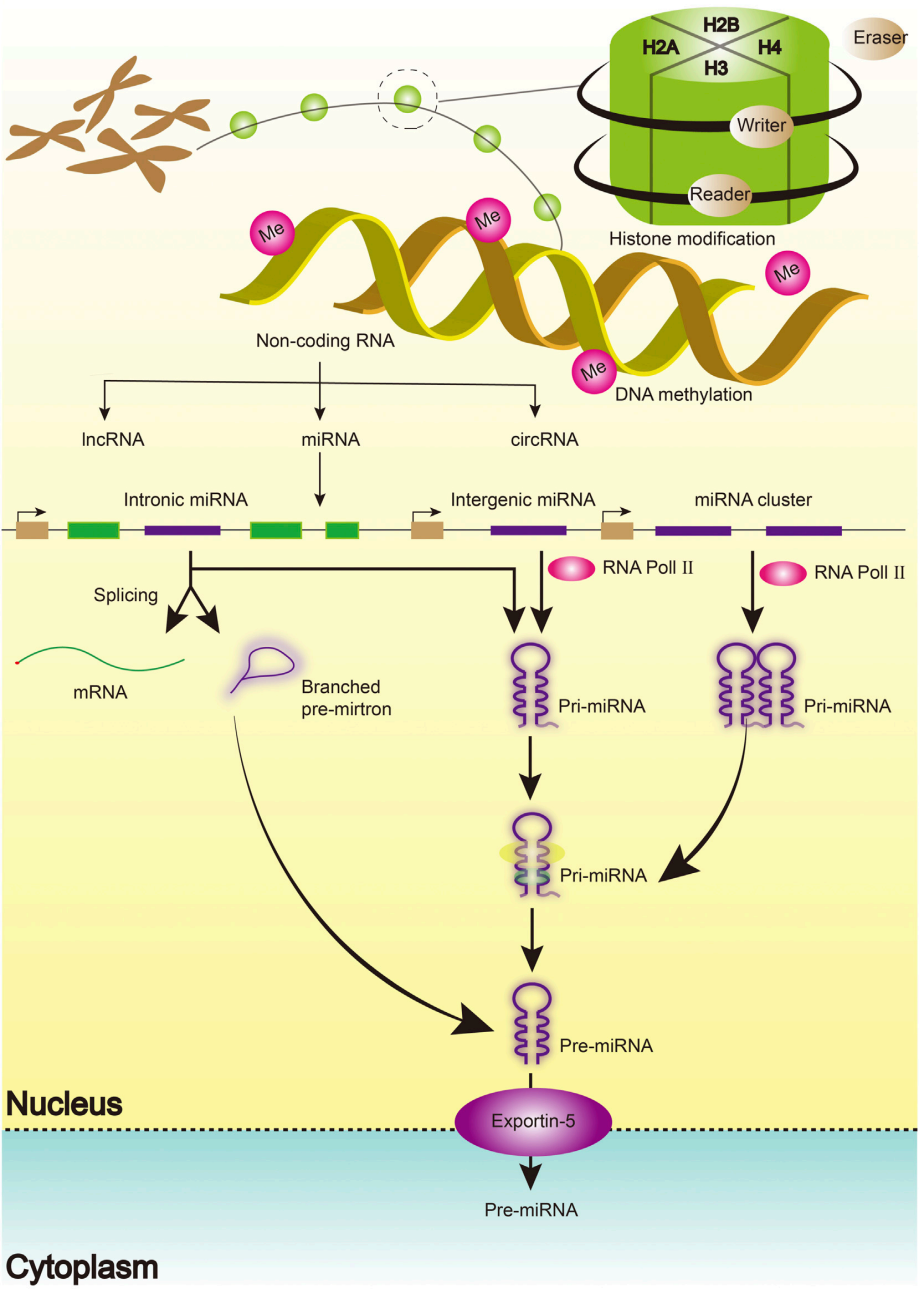
The Biogenesis of miRNAs.

Emerging evidence has defined the biogenesis, mechanism of action, and function of miRNAs. miRNAs are single-stranded, short, non-coding RNA molecules that regulate gene expression at the post-transcriptional level by binding to mRNAs (Smolarz et al., 2022). Similarly, miRNAs coordinate key cellular processes, including cell differentiation, angiogenesis, migration, apoptosis, and oncogenesis (Shan et al., 2022; Smolarz et al., 2022). A single miRNA has been shown to influence thousands of genes by identifying complementary sequences at the target mRNA’s 3ʹUTR end. The process begins with the transcription of miRNA genes in the nucleus as lengthy pri-miRNAs with a 5ʹ cap and a 3ʹ polyA tail, which are then cleaved into 70 nt hairpin precursor miRNAs by a microprocessor complex composed of Drosha and DGCR8 proteins (pre-miRNA). It is then cleaved into 22-bp miRNA/miRNA duplexes by the Dicer/TRBP enzyme complex. Finally, the miRNA/miRNA duplexes separate, leaving one of the strands bound to an Argonaute (AGO) protein, resulting in a mature functional miRNA with a length of 20–25 nucleotides (Figure 1).

Furthermore, miRNAs can be detected in human cell lines and bodily fluids, such as blood, urine, and cerebrospinal fluid, and miRNA signatures unique to various disorders have been discovered in tissues and bodily fluids (Weber et al., 2010). Using quantitative polymerase chain reaction (qPCR), fluorescence *in situ* hybridization (FISH), or next-generation sequencing (NGS), differentially expressed miRNAs can be profiled in a variety of specimens (Cao et al., 2021; Paulsen et al., 2021). Previous studies have shed light on the epigenetic mechanisms that play a role in many illnesses and could lead to the development of new diagnostic and therapeutic tools.

### MicroRNAs as regulators of kidney homeostasis

Numerous studies have confirmed that miRNAs play critical roles in renal development and homeostasis and that changes in miRNA expression are extremely relevant to disease processes. miRNAs were more abundant in the kidneys than in other tissues. Microarray analysis of human and mouse kidneys revealed the expression of miR-192, miR-194, miR-204, miR-215, miR-216, miR-146a, and miR-886. Additionally, miRNAs such as let-7a-g, miR-10a/b, miR-21, miR-30a-e, miR-130, miR-143, miR-196a/b, miR-200a, miR-23, and miR-872 are expressed in the renal tissue (Chandrasekaran et al., 2012; Caus et al., 2021).

miRNAs play a critical role in the maintenance of renal homeostasis. MiR-193a-5p is required for the differentiation of glomerular epithelial cells during embryogenesis. Both glomerular parietal epithelial cells (PECs) and podocytes develop from the same mesenchymal cells during development (Jha et al., 2020). Moreover, the expression of miR-193a-5p affects whether cells take on the phenotype of PECs or podocytes. MiR-26a was the most abundant miRNA in podocyte cytoplasm at particularly high levels (Li et al., 2019). MiR-26a expression in the glomerulus was positively correlated with *Podxl, Synpo*, *Cd2ap, Myh9, Acta2,* and *Vim* but negatively correlated with albumin-to-creatinine (ACR) ratio. Each of these targets is directly associated with the organization of actin protein synthesis in the cytoskeleton, which is required for podocyte function; consequently, they are also associated with glomerular dysfunction. Furthermore, miRNAs play essential roles in podocytes. Dicer converts pre-miRNAs into mature miRNAs (Hejazian et al., 2020). In Dicer-deficient mice, proteinuric renal disease and collapsing glomerulopathy (CG) with glomerular and tubulointerstitial fibrosis and renal failure are caused by the loss of miR-30 family members from podocytes (Gallagher et al., 2010).

It is clear that miRNAs form a vital part of the regulatory functions in renal development, maintenance of kidney functions, and progression of kidney diseases (Trionfini et al., 2015). Sun et al. (2004) discovered that miR-192 has a transcription factor-binding site for the proto-oncogene ETS-1, which is important for normal kidney development and maintenance of glomerular integrity in mammals. Similarly, studies of ETS-1 knockout mouse kidneys have demonstrated various glomerular abnormalities, including sclerosis, atrophy, and immature glomeruli. Bai et al. (2011) demonstrated that overexpression of miR-335 and miR-34a induces premature senescence in young mesangial cells by suppressing superoxide dismutase 2 (SOD2) and thioredoxin reductase 2 (Txnrd2), increasing reactive oxygen species (ROS). In addition to early renal development, miRNAs also participate in renal senescence. Antisense miR-335 and miR-34a inhibited mesangial cell senescence by increasing SOD2 and Txnrd2 levels and decreasing ROS levels (Bai et al., 2011). Numerous studies have established that dysregulation of miRNAs is associated with various kidney diseases (Sun et al., 2004; Bai et al., 2011; Trionfini et al., 2015).

### MicroRNAs in lupus nephritis

Numerous reviews and preclinical and clinical studies have established and discussed the critical roles of various miRNAs in the development, prognosis, and therapies of LN (Dominguez-Gutierrez et al., 2014; Habibi et al., 2016; Li et al., 2017; Trinh et al., 2017; Zhu et al., 2017; Xu et al., 2018; Ye et al., 2018; Feng et al., 2019; Li et al., 2019; Liu et al., 2019; Qi et al., 2020; Shao et al., 2020; Sun et al., 2020; Abdul-Maksoud et al., 2021; Du et al., 2021; Perez-Hernandez et al., 2021; So et al., 2021; Xu et al., 2021; Zhang et al., 2021). There has been much research and review on the role of miRNAs in LN over the last decade, and several well-known miRNAs have been linked to the development and progression of LN (Table 1).

**TABLE 1:** miRNAs involved in the development and progression of lupus nephritis.

MiRNAs	Pathway	Influence on key elements in the pathogenesis	References
miR-146b-5p	JAK1/STAT1 pathway	Inflammation	Ye et al. (2018)
miR-124	TRAF6	Inflammation	Ye et al. (2018)
miR-146a	TLR/IFN	Inflammation	Li et al. (2017)
miR-199a	Klotho/NF-KB	Inflammation	Zhang et al. (2021)
miR-27b-3p	STING/IRF3/IFN-I	Inflammation	So et al. (2021)
miR-183	TGF-β/Smad/TLR3 pathway	Fibrosis	So et al. (2021)

Inflammation is a protective mechanism triggered in response to pathological conditions to maintain cellular homeostasis and integrity. Multiple studies have shown that many miRNAs either use or control the IFN/nuclear factor kappa B (NF-κB).

**FIGURE 2:**
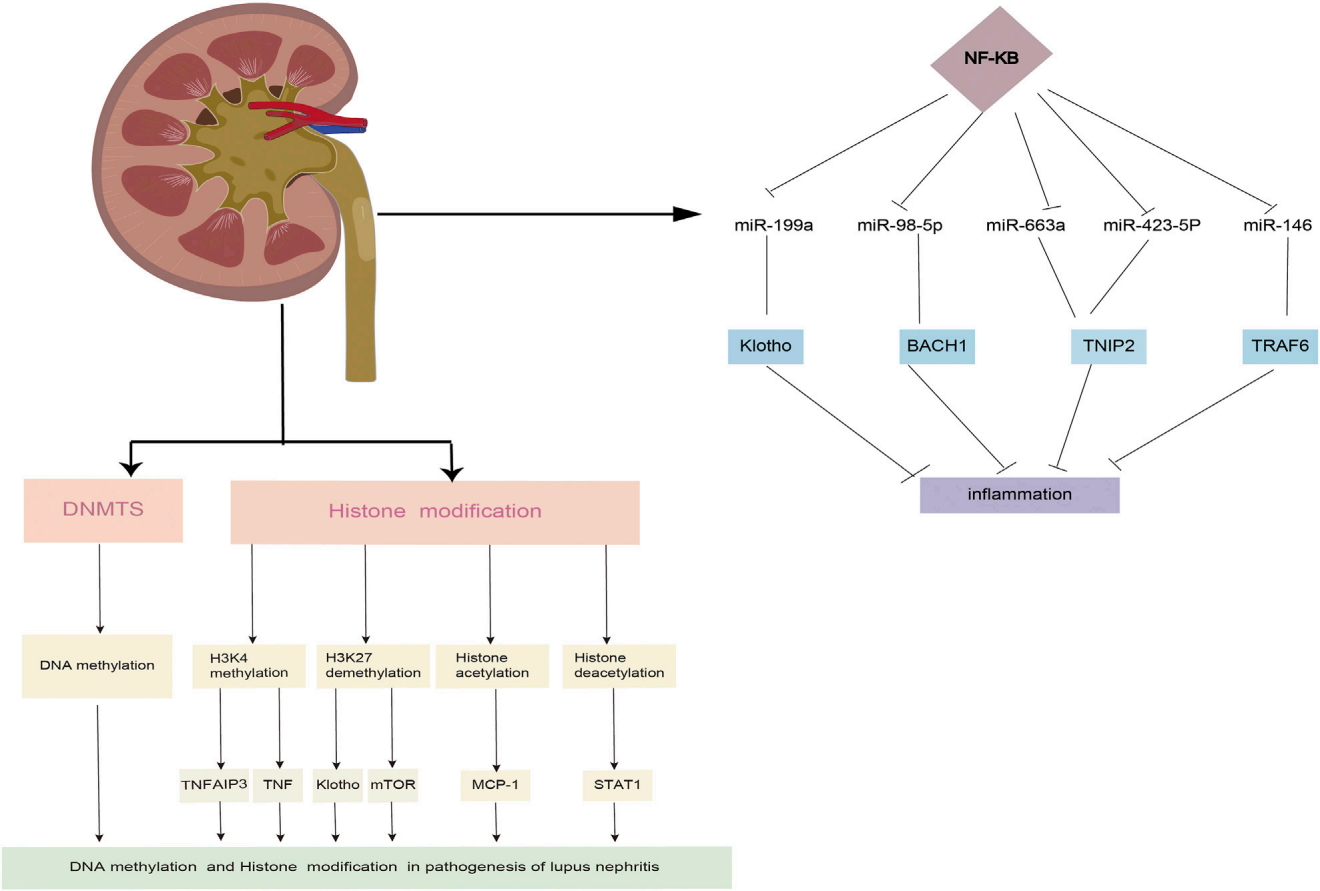
The roles of DNA methylation and histone modifification in the LN process.

Inflammatory pathways, and that changes in the expression of these miRNAs may be linked to inflammation in LN (Feng et al., 2019; Shao et al., 2020; Du et al., 2021). The critical role of macrophages in inflammatory processes has been well established. A study revealed that miR-98-5p elicits macrophage transformation into the M2 phenotype by directly inhibiting the expression of BTB and CNC homology 1 (BACH1) (Du et al., 2021). In addition, studies also discovered that BACH1 helps cells grow and produce inflammatory factors through NF-κB (Liu et al., 2019; Sun et al., 2020). Ye et al. (2018) found that miR-199a can directly control the activation of NF-κB, which is important for protecting the kidneys from damage. Through the transcriptional regulation of inflammatory factors, the NF-κB signaling pathway is intimately linked to the initiation and progression of LN. A previous study confirmed that miR-663a/miR-423-5p fundamentally contributed to lipopolysaccharide (LPS)-induced NF-κB activation by targeting TNIP2 (Li et al., 2017). Several studies have suggested that miR-146a is typically activated by Toll-like receptor (TLR) activation *via* LPS stimulation and plays an important role in the regulation of the NF-κB and type I interferon (IFN) pathways by targeting signal transducers, such as TNF receptor-associated factor 6 (TRAF6), IL-1 receptor-associated kinase 1 (IRAK1), and IFN regulatory factor 5 (IFN-5). Furthermore, miR-124 is a significant diagnostic biomarker for active LN (Feng et al., 2019; Shao et al., 2020) (Figure 2).

miR-146b-5p targets IFI35 to inhibit inflammatory reactions and apoptosis *via* inactivation of the JAK1/STAT1 pathway, providing a novel mechanism for treating LN (Zhang et al. (2021). Found that miR-124 can also inhibit the growth and inflammation of renal mesangial cells by targeting TRAF6 in the same way (Li et al., 2019; So et al., 2021). Xu et al. (2021) recently investigated the role of miR-27b-3p in modulating STING/IRF3/IFN-I signaling in LN in a similar manner. Plasma samples were collected from patients with LN and kidney tissue samples from nephropathic mice. They also performed an *in vitro* evaluation using HK-2 cells. CircELK4 contributed to renal injury by promoting the inflammatory response and cell damage in LN by acting as an miR-27b-3p sponge (Xu et al., 2021).

miR-146a can downregulate TRAF6 and IRAK1 expression to terminate the inflammatory response of glomerular mesangial cells (Zhu et al., 2017). Upregulation of miR-146a significantly inhibits the expression of interleukin-6 and TNF-a. In addition, miR-146a can significantly reduce the expression of inflammatory factors (RELA, IRAK1, and IL-1B) in kidney tissues, thus prolonging the survival time of SLE-prone mice (MRL/lpr mice) (Habibi et al., 2016). Furthermore, miR-146a inhibits TLR-induced type I IFN production and myeloid differentiation factor 88 (MyD88) pathway activation (Trinh et al., 2017). MiR-146a may lose its function in patients with SLE and be unable to constrain STAT1. Furthermore, STAT1 has two subtypes (STAT1a and STAT1b). STAT1a is the long isoform predicted by TargetScan to be regulated by miR-146a, whereas STAT1b is not anticipated to be regulated by miR-146a (Dominguez-Gutierrez et al., 2014). These findings indicate that miR-146a plays a beneficial role in preventing LN progression.

Renal fibrosis is a common complication in most kidney diseases, including LN. Huang et al. (2020) investigated the role of miR-133 in the initiation and progression of fibrosis in LN (Perez-Hernandez et al., 2021). Once again, samples were obtained from patients with nephropathy, and an *in vitro* study using cultured kidney cells was conducted. They found that miR-133 directly inhibited LASP1 expression. They also found that miR-133 suppresses proliferation and promotes apoptosis through its binding with LASP1 in human mesangial cells. Qi et al. (2020) discovered a novel mechanism of miR-183 regulation in nephropathy. They investigated miR-183 expression in human glomerular endothelial cells (HRGECs) and MRL/lpr mice. miR-183 expression was significantly lower in MRL/lpr mice with lupus, whereas increased miR-183 expression inhibited renal fibrosis and inflammation in mice with LN. They discovered that miR-183 suppresses TGFBR1 expression by directly disrupting the TGF-/Smad/TLR3 pathway, thereby repressing renal fibrosis. Additionally, they demonstrated that oxymatrine treatment significantly inhibited fibrosis of renal tissue caused by miR-183 antagonists (Qi et al., 2020).

Further studies have demonstrated that miRNAs are crucial in diagnosing, monitoring disease activity, prognosticating, and treating LN in clinical settings. MiR-181a and miR-223 expression analysis in combination may be useful for diagnosing SLE and predicting LN in Egyptians (Xu et al., 2018; Abdul-Maksoud et al. (2021). demonstrated, for the first time, that miR-181a has a significant positive correlation with SLEDAI, proteinuria, blood urea, and serum creatinine (*p* < 0.001), whereas miR-223 downregulation is significantly negatively correlated with SLEDAI, proteinuria, blood urea, and serum creatinine (Abdul-Maksoud et al., 2021). Another study reported a significant negative correlation between miR-125a expression and SLEDAI-2K (*p* < 0.01) and SLICC (*p* < 0.01) levels in patients. Additionally, it was significantly inversely related to the patients’ ESR and proteinuria (Xu et al., 2018).

Furthermore, miR-125a expression has been linked to a significant decrease in disease activity, inflammation, and IL-17 levels in patients with SLE (Eissa et al., 2021). Similarly, Cai et al. (2019) demonstrated that miRNA-145 is associated with the pathogenesis of renal vascular lesions and may be a potential treatment for children with LN (Eissa et al., 2021). Additionally, they discovered that as vascular damage increased in patients with LN, miR-145 expression decreased. Abnormal levels of miR-21, miR-150, and miR-423-3p, which may be associated with renal dysfunction, were detected in plasma samples from patients with LN. Additionally, miR-26a overexpression significantly decreased the expression of two LN biomarkers: monocyte chemotactic protein (MCP)-1 and vascular cell adhesion protein 1 (VCAM-1). Thus, miR-26a can be used as a potential LN biomarker (Li et al., 2019).

## Introduction to epigenetics

In contrast to genetic modifications, epigenetic modifications primarily include but are not limited to DNA methylation, histone modifications, and miRNA regulation, all of which are functionally related. Epigenetic changes are not only important for normal bodily function but also play a major role in the pathogenesis and development of many diseases, especially LN. DNA methylation is catalyzed by a family of enzymes known as DNA methyltransferases (DNMTs), whose primary function is to transfer a methyl group from S-adenyl methionine to cytosine at position C5, yielding 5-methylcytosine (5-mC) (Hu et al., 2021). Several studies have shown that different DNMTs may play diverse roles in the methylation of DNA (Wu and Zhang, 2014; van der Harst et al., 2017; Guo et al., 2019). For example, DNMT1 is involved in maintaining pre-existing methylation patterns during DNA replication, whereas DNMT3A and DNMT3B catalyze *de novo* methylation (Srut, 2021; Joudi et al., 2022). In contrast, active DNA demethylation is regulated by TET genes that oxidize 5-mC (Srut, 2021). A consistent methylation pattern is required for the proper functioning of many cellular and biological processes, including reproduction and differentiation. It is also required for proper embryonic development (Lee and Kim, 2021).

Histones are composed of core histone proteins (H2A, H2B, H3, and H4) and linker histones (H1 and H5) that interact with DNA and other histones to form higher-order chromatin structures (Zanger et al., 2018; Colpaert and Calore, 2021) (Figure 1). The nucleosome, which is composed of DNA and wrapped histone proteins, is regarded as the fundamental unit of chromatin (Shao et al., 2021). Each core histone subunit is found twice in the nucleosome and has an amino-terminal tail protruding from it. Most histone post-translational modifications have been described, including methylation, acetylation, glycosylation, ubiquitination, SUMOylation, and ADP-ribosylation (Chen et al., 2017). There are four types of modifications, namely histone acetyltransferase, histone methyltransferase (HMT), histone deacetylase (HDAC), and histone demethylase (HDM), in amino acid residues of histone. HDACs have been implicated in the regulation of protein acetylation, which is implicated in various pathophysiological states. HDAC inhibitors play an important role in anti-cancer therapy (van der Harst et al., 2017). As a novel DNMT inhibitor, MG98 may enable the re-expression of tumor suppressor genes and inhibit tumor growth (Zwergel et al., 2019; Zwergel et al., 2020).

### Epigenetics and lupus nephritis

LN has a complex multifactorial etiology, including genetic, epigenetic, environmental, and physiological factors. Furthermore, changes in DNA methylation have been proposed as diagnostic markers that may be able to predict specific disease manifestations in LN Coit et al. (2020). conducted methylation quantitative trait loci (meQTL) analyses, which revealed that methylation changes correlate with disease activity and progression of LN throughout the course of the disease (Coit et al., 2020). Additionally, they identified a single CpG site associated with the association between DNA methylation levels and effective nephritis. The group also examined the genetic differences in the proportion of CpG sites that were differentially methylated in African American and European American patients with lupus who underwent ancestral meQTL analysis (Coit et al., 2020).

Differential methylation was found in whole-genome DNA methylation and gene expression arrays performed by Zhu et al. (2016) in peripheral blood mononuclear cell samples from patients with SLE with or without LN. After genome-wide methylation analysis, Mok et al. (2016) discovered that hypomethylation of CpG sites in or near tissue hypoxia and interferon signaling response genes is associated with LN. Similarly, epigenome-wide association studies (EWAS) revealed a link between DNA methylation, SLE risk, and disease heterogeneity (Lanata et al., 2018). Wardowska et al. (2019) recently published a clinical report based on previous research on the relationship between DNA methylation status and LN. Zhao et al. (2018) demonstrated that the downregulation of BDH2 promotes DNA demethylation in CD4^+^ T cells in SLE (Dai et al., 2016). DNMT1 and MBD2 expression was decreased in myeloid dendritic cells (mDCs) isolated from patients with SLE. Notably, patients with moderate LN had decreased DNMT1 expression and increased MBD2 expression in mDCs, whereas patients with mild LN showed a declining tendency for both genes associated with mDC epigenetic status (Wardowska et al., 2019). Additionally, Li et al. (2018) discovered that RCAN1 RNA expression was decreased in glomeruli in mouse models of HIV-associated nephropathy and diabetic nephropathy as well as in human LN. Therefore, DNA methylation status may prove to be a valuable prognostic marker for LN prognosis.

Post-translational modifications of histones also contribute significantly to the expression of genes associated with LN progression. Zheng et al. (2020) demonstrated that trichostatin A could significantly inhibit *cd40l* gene expression, decreasing *IL10* gene expression and increasing *IFN-γ* gene expression in SLE. STAT3, which is overexpressed in T cells from patients with SLE, plays a critical role in epigenetic remodeling *via* interactions with histone acetyltransferase p300 (Hedrich et al., 2014; Hedrich, 2017). Further, HDAC2, HDAC7, and SIRT1 levels decreased, whereas HDAC6, CREBBP, and PCAF levels increased (Regna et al., 2015; Yang et al., 2015; Regna et al., 2016). It is well established that TNF-α-induced protein 3 (TNFAIP3) plays a role in controlling inflammatory responses during LN development. H3K4me3 levels in the promoter region of the gene were significantly associated with TNFAIP3 expression. This is because the downregulation of TNFAIP3 may have been regulated by histone H3K4 demethylation, which results in a decreased amount of H3K4me3 in the promoter region of the *TNFAIP3* gene (Yang et al., 2015; Zhao et al., 2017). In addition, compared to control samples, the expression of Let-7 miRNAs was markedly upregulated, while the TNFAIP3 level was noticeably downregulated (Liu et al., 2015; Honarpisheh et al., 2018). A recent study discovered that H3K27me3 regulates Klotho expression in the kidneys of elderly mice. Aging plays a critical role in renal tubules by increasing H3K27me3 levels and decreasing the hyperphosphorylation of Klotho and mTOR (Han and Sun, 2020) (Figure 2). EZH2-mediated epigenetic modifications are required for allogeneic T-cell-induced lupus disease (Scharer et al., 2020; Zhen et al., 2020). The most significant benefit of 3-DZNep is its ability to inhibit EZH2 in MRL/lpr mice, which can lessen renal damage and improve survival rates (Rohraff et al., 2019; Scharer et al., 2020). When mice received 3-DZNep treatment, their ACR remained steady, with marked reduction in their glomerulonephritis and crescent development (Tsou et al., 2019; Li et al., 2021) (Table 2).

**TABLE 2:** Epigenetics on LN in animal models.

Epigenesis	Animal model	Effects and mechanisms	References
Histone deacetylase	Lupus-prone mice	Isoform-selective HDAC inhibitors decrease the expression of HDAC6 and acetylate critical signaling and transcription factors in inflammation	Regna et al. (2015)
Histone deacetylase	NZB/W mice	HDAC6 inhibition decreased SLE disease by inhibiting immune complex-mediated glomerulonephritis, sera anti-dsDNA levels, and inflammatory cytokine production and increasing splenic Treg cells	Regna et al. (2015)
Histone methyltransferase	Murine bm12 model of lupus-like chronic graft versus host disease (cGVHD)	EZH2 inhibition suppresses autoantibody production and GC formation in bm12 lupus-like cGVHD and decreases affinity maturation and isotype switching in response to immunization with a T cell-dependent antigen	Zhen et al. (2020)
Histone deacetylase	MRL/*lpr* mice	Inhibiting EZH2 with DZNep treatment before or after disease onset improved survival and reduced anti-dsDNA antibody production	Rohraff et al. (2019)
DNA hydroxymethylation	MRL/*lpr* mice	inhibiting miR-21 *in vivo* improves intracellular iron homeostasis and inhibits Tfh cell overexpansion	Gao et al. (2022)
DNA demethylation	Lupus-Prone *lpr* Mice	DNT cells manifest discrete sites of extensive demethylation throughout the genome, and these sites correspond to the location of a large proportion of the upregulated genes	Scharer et al. (2020)
DNA methylation	NZB/NZW F1 mice	EZH2 inhibition has the potential to inhibit the IFN-I signaling pathway and alleviate lupus nephritis	Wu et al. (2014)
DNA hypomethylation	MRL-*lpr* lupus mice	DNA methylation regulates genomic imprinted DLK1-Dio3 miRNAs in autoimmune lupus	Dai et al. (2016)
DNA hypomethylation	cKO mice	Downregulation of BDH2 modulates iron homeostasis and promotes DNA demethylation in CD4^+^ T cells of systemic lupus erythematosus	Zhao et al. (2017)

### Environmental factors and epigenetic regulations in lupus nephritis

Numerous lifestyle factors have been shown to alter DNA methylation patterns, including food intake, obesity, physical activity, cigarette use, alcohol use, environmental contaminants, and psychological stress (Lanata et al., 2018; Stover et al., 2018). For instance, the DNMT and TET enzyme families are directly affected by environmental pollutants. Chemicals may also affect the availability of S-adenosylmethionine, which is the source of gene-specific DNA methylation patterns (Martin and Fry, 2018). Increased oxidative stress has been linked to ERK signaling pathway deregulation, which results in DNMT1 downregulation and global DNA demethylation in T cells in patients with LN (Li et al., 2014; Joudi et al., 2022). ROS are produced by environmental stressors such as UV light, cigarette smoke, silica, and others, which may cause DNA methylation abnormalities by interfering with the MAPK/ERK pathway (Somers and Richardson, 2014).

Growing evidence suggests that specific adverse health outcomes are “programmed” during pregnancy and the first few months after birth, most likely by altering DNA methylation patterns (Tsokos et al., 2016; Martin and Fry, 2018). Epigenetic dysregulation caused by environmental chemicals in primordial germ cells, embryos, and fetuses can have various negative effects. Prenatal vanadium exposure, for example, has been associated with changes in DNA methylation of the interleukin 4 (*IL4*) and *INF-γ* genes. (Dorgham et al., 2015; Martin and Fry, 2018). Although DNA methylation aberrations have been linked to prenatal environmental exposure in numerous studies, it is still unclear how these aberrations contribute to LN.

## Epigenetics and microRNAs interplay

Emerging topics, such as epigenetics and miRNAs, can potentially transform the therapeutic landscape. The expression of several miRNAs, including miR-375, miR-29, and miR-34, is epigenetically regulated *via* methylation and histone modifications during various pathological processes. In contrast, miRNAs such as miR-449a, miR-148, miR-101, miR-214, and miR-128 regulate various epigenetic enzymes that play a role in disease progression (Poddar et al., 2017). Many developmental and tissue remodeling processes, including the epithelial-mesenchymal transition (EMT), are epigenetically maintained by miR-200c/141, which is regulated by DNA methylation. Numerous research studies and reviewers have focused their attention recently on the interaction between miRNAs and epigenetics, particularly LN. Additionally, miRNAs play a critical role in epigenetics by directly interacting with key enzymes involved in epigenetic processes (Iorio et al., 2010). As a result of DNA methylation and histone modification in the regulatory regions of miR-142, CD4^+^ T-cell activation and B-cell hyperstimulation in SLE are reduced (Ding et al., 2012; Frangou et al., 2013). This demonstrates the existence of a regulatory loop that links miRNA expression to epigenetic modification (Osella et al., 2014).

According to various reports, the associations between miRNAs and epigenetic enzymes that regulate various diseases are relatively prominent. The first evidence indicates that the miR-29 family directly regulates the *de novo* DNA methyltransferases DNMT-3A and -3B (Iorio et al., 2010). In adult neural stem cells (aNSCs), MeCP2, a DNA methyl-CpG-binding protein, has been shown to epigenetically regulate specific miRNAs, namely aNSCs (Szulwach et al., 2010). Neurodevelopmental disorders such as Rett syndrome result from MeCP2 *de novo* mutations. Szulwach et al. (2010) discovered that miR-137 regulates aNSC proliferation and differentiation *in vitro* and *in vivo* by directly targeting MeCP2. These findings demonstrate that neurogenesis is regulated by connections between epigenetic modifications and the miRNA pathway (Jobe et al., 2012). Similar reactions have been reported in patients with impaired angiogenic responses. A study has established that miR-30a-MeCP2-SIRT1 dysregulation results in dysregulated endothelial angiogenic responses. Specifically, various miRNAs connect to DNMTs and play a role in the pathophysiology of various diseases (Sun et al., 2013; Volkmann et al., 2013).

Epi-miRNA is a type of miRNA that regulates epigenetic machinery, including DNA methyltransferases. A previous study showed that miR-148a/b, miR-152, miR-301, and miR-302 could modulate DNMTs, while others, such as the miR-29 family, modulate DNMT-3a and DNMT-3b (Poddar et al., 2017). In addition to DNA methylation enzymes, miRNAs regulate histone modifiers, as demonstrated by miR-449 and miR-1, which modulate HDAC1 and HDAC4, respectively. We conducted a comprehensive literature review to establish a feedback connection between miRNA modulation and epigenetic modifications in LN (Table 3).

**TABLE 3:** The Interplay between epigenetics and miRNA.

Regulator	Target	Function	References
MiR-21	DNMT1	MiR21-RASGPR1-DNMT1-Ras-MAPK pathway, promote cell hypomethylation	Pan et al. (2010)
MiR-148	DNMT1	Promote cell hypomethylation	Pan et al. (2010)
MiR-29b	DNMT1	MiR-29b-sp1-DNMT1, lead to DNA hypomethylation	Qin et al. (2013)
MiR-30a	MeCP2	MiR-30a-MeCP2-SIRT1, dysregulation in endothelial angiogenic responses	Volkmann et al. (2013)
MiR-101	EZH2	Negatively correlated with lupus disease activity	Tsou et al. (2018)
MiR-26a	EZH2	Negatively correlated with lupus disease activity	Tsou et al. (2018)
HDAC3	MiR-30d	Podocyte cytoskeleton rearrangement and apoptosis	Liu et al. (2015)
DNA methylation	Dlk1-Dio3 miRNAs	A positive correlation between DNA hypomethylation and upregulation of DLK1-Dio3 miRNA	Dai et al. (2016)

### Epigenetic modification regulating microRNAs expression in lupus nephritis

Over the last few years, the effects of epigenetic modifications regulating miRNAs in LN have gained increasing attention. Liu et al. (2016) demonstrated that HDAC3 plays a vital role in suppressing miR-30d expression and podocyte injury. TGF-induced repression of miR-30d *via* the Smad2/3-HDAC3-NCoR repression complex also resulted in podocyte cytoskeleton rearrangement and apoptosis. To verify the role of HDAC in miR-30d suppression, the HDAC inhibitor trichostatin A was used. It did not affect miR-30d promoter transcription in isolation, but when combined with TGF, it reversed the TGF-induced suppression of the miR-30d promoter (Shi et al., 2013; Wu et al., 2014; Liu et al., 2016). The researchers concluded that DNA methylation regulates the expression of Dlk1-Dio3 miRNAs in SLE (Dai et al., 2016; Dai et al., 2021). Dai et al. (2016) discovered that 5-Aza-2′-deoxycytidine, a specific DNA methylation inhibitor, dramatically enhanced the expression of DLK1-Dio3 miRNAs. The epigenetic modulator EZH2 may be necessary for shifting the epigenetic landscape in patients with lupus and with increased disease activity (Coit et al., 2016). Zhao et al. (2016) established that EZH2 overexpression leads to the methylation of junctional adhesion molecule A (JAM-A), which might promote T cell migration in patients with lupus (Table 3).

### MicroRNAs modulating epigenetic machinery in lupus nephritis

miRNAs influence the onset and progression of LN in various ways, one of which is the alteration of epigenetic switches. Some lupus-associated miRNAs control DNA methylation by targeting the DNA methylation enzymes or proteins involved in the methylation process. Pan et al. (2010) discovered that miR-21 and miR-148a are overexpressed in patients with lupus and promote cell hypomethylation by directly targeting DNA methyltransferase 1 (DNMT1). Further studies have demonstrated that miR-21 effectively downregulates *DNMT1* transcription by inhibiting *RASGRP1*, an essential autoimmune gene that mediates the Ras–MAPK pathway upstream of DNMT1 (Pan et al., 2010). MiR-148a is upregulated in lupus T cells and directly targets DNMT1, whereas miR-29b is upregulated and indirectly targets DNMT1 (Zhao et al., 2011). A study found that miR-29b repressed SP1, a positive modulator of DNMT1, in lupus T cells, leading to DNA hypomethylation (Zhao et al., 2011; Qin et al., 2013). Insufficiency of miR-142-3p/5p was correlated with greater histone protein modification and DNA hypermethylation in the regulatory region of the miR-142 precursor in lupus (Ding et al., 2012; Zununi Vahed et al., 2018). Navarro Quiroz et al. (2019) discovered a favorable correlation between SUV39H2 mRNA and H3K9 methylation levels. The suppressor of SUV39H2 is the histone methyltransferase necessary to methylate histones H3K9, therefore repressing or silencing target genes (Navarro Quiroz et al., 2019). Additionally, miR-101 and miR-26a have been shown to be adversely associated with lupus disease activity in CD4^+^ T cells of patients with lupus (Moulton and Tsokos, 2015; Tsou et al., 2018) (Table 4).

**TABLE 4:** miRNA expression or alteration in epigenetics on LN.

miRNA	Epigenetic	Effects	References
miR-98-5p		Elicit macrophage transformation into the M2 phenotype by directly inhibiting the expression of BTB and CNC homology 1 (BACH1) inhibit secretion of TNF-α and IL-6	Du et al. (2021)
miR-199a		Regulate the transcription activation of NF-κB by directly targeting Klotho	Ye et al. (2018)
miR-146b-5p		Target IFI35 to inhibit inflammatory reactions and apoptosis *via* inactivation of the JAK1/STAT1 pathway	Zhang et al. (2021)
miR-124		Suppress the expression of TRAF6 through direct binding to the 3′-UTR of mRNA	Zhang et al. (2019)
miR-27b-3p		Contribute to LN by directly binds STING mRNA, CircELK4 by acting as a miR-27b-3p sponge to regulate STING/IRF3/IFN-I signaling	Xu et al. (2021)
miR-146a		Regulate inflammatory response by suppressing the IRAK1/TRAF6/LPS pathway to ameliorate lupus nephritis activity	Perez-Hernandez et al. (2021)
miR-183		Inhibit the expression of Tgfbr1 by direct targeting to disrupt the TGF-β/Smad/TLR3 pathway, thus repressing renal fibrosis and the secretion of inflammatory factors in LN.	Qi et al. (2020)
miR-181a miR-223		miR-181a has a significant positive correlation with SLEDAI, proteinuria, blood urea, and serum creatinine (*p* < 0.001), whereas miR-223 downregulation was significantly negatively correlated with SLEDAI, proteinuria, blood urea, and serum creatinine	Abdul-Maksoud et al. (2021)
miRNA-145		Involve in the pathogenesis of renal vascular lesions and may be a potential treatment for children with LN	Cai et al. (2019)
	RCAN1	RCAN1 RNA expression was decreased in glomeruli in mouse models of HIV-associated nephropathy and diabetic nephropathy as well as in human LN	Li et al. (2018)
	Histone acetyltransferase p300	STAT3 plays a critical role in epigenetic remodeling *via* interactions with histone acetyltransferase p300. Stat3 promotes IL-10 expression in lupus T cells through trans-activation and chromatin remodeling	Hedrich et al. (2014)
	HDAC6	Increased HDAC6 expression and activity contribute to SLE pathogenesis, and isoform-selective HDAC inhibitors may prove beneficial in the treatment of SLE by acetylating key signaling and transcription factors in inflammation and cell activation	Regna et al. (2015)
	EZH2	EZH2-mediated epigenetic modifications are required for allogeneic T-cell-induced lupus disease	Zhen et al. (2020)
	H3K4me3	TNFAIP3 may have been regulated by histone H3K4 demethylation, which results in a decreased amount of H3K4me3 in the promoter region of the TNFAIP3 gene	Zhao et al. (2017)
	T Cell DNA Methylation	Our findings indicate that oxidative stress may contribute to human lupus flares by inhibiting ERK pathway signaling in T cells to decrease. DNMT-1 and cause DNA demethylation	Li et al. (2014)
miR-21	BDH2	Promote DNA demethylation in CD4^+^ T cells through inhibiting BDH2 expression	Zhao et al. (2017)
miR-21 miR-148a	DNMT1	Promote cell hypomethylation by directly targeting DNA methyltransferase 1 (DNMT1) miR-21 indirectly downregulated DNMT1 expression by targeting an important autoimmune gene, RASGRP1, which mediated the Ras–MAPK pathway upstream of DNMT1; miR-148a directly downregulated DNMT1 expression by targeting the protein coding region of its transcript	Pan et al. (2010)
miR-126	DNMT1	miR-126 directly inhibits DNMT1 translation. The overexpression of miR-126 caused the demethylation and up-regulation of genes encoding CD11a and CD70, thereby causing T cell and B cell hyperactivity	Zhao et al. (2011)
miR-29b	DNA hypomethylation	Contribute to DNA hypomethylation of CD4^+^ T cells in SLE by indirectly targeting DNA methyltransferase 1. Overexpression of miR-29b in CD4^+^ T cells from healthy donors led to the DNA hypomethylation and up-regulation of genes encoding CD11a and CD70	Qin et al. (2013)
miR-26a miR-101	EZH2	MiR-26a and miR-101 downregulated EZH2, and were reduced in lupus CD4^+^ T cells. Overexpressing EZH2 in CD4^+^ T cells resulted in significant DNA methylation changes	Tsou et al. (2018)

## Conclusion

The mutual relationship between epigenetics and miRNA in the physiological and pathophysiological processes of LN has garnered significant attention from researchers in recent years. From the above discussion and evidence, it is clear that miRNAs are becoming increasingly critical in controlling mechanisms implicated in the regulation of gene expression. Additionally, epigenetic mechanisms, including DNA methylation and histone modification, are critical for LN development. However, our knowledge of the epigenetic mechanisms occurring in LN is currently limited. This review emphasizes the interaction between DNA methylation and histone modifications and their functions in LN. In addition to DNA methylation, histone modification, and non-coding RNA, RNA methylation is required for post-translational modification of messenger RNA. However, the functions of epigenetic alterations are not completely understood. Recently, single-nucleus ATCT-seq in combination with snRNA-seq was utilized to determine cell type-specific chromatin accessibility, which would aid in narrowing the differently accessible knowledge of kidney cell heterogeneity (Muto et al., 2021; Shao et al., 2021). Additional in-depth studies of RNA methylation may aid in the clarification of SLE pathophysiology and provide new insights into diagnostic and treatment techniques.
